# Influence of Normobaric Hypoxia on Maximal Force Production Following High-Intensity Resistance Circuit Training

**DOI:** 10.3390/jfmk11010098

**Published:** 2026-02-27

**Authors:** Ismael Martínez-Guardado, Diego A. Alonso-Aubin, Juan Hernández-Lougedo, Domingo J. Ramos-Campo

**Affiliations:** 1 LFE Research Group, Department of Health and Human Performance, Faculty of Physical Activity and Sport Science (INEF), Universidad Politécnica de Madrid, Calle de Martín Fierro, 7, 28040 Madrid, Spain; domingojesus.ramos@upm.es; 2 Strength Training and Neuromuscular Performance Research Group (STreNgthP), Faculty of Health Sciences—HM Hospitals, University Camilo José Cela, C/Castillo de Alarcón, 49, Villanueva de la Cañada, 28692 Madrid, Spain; diegoalexandre.alonso@ucjc.edu (D.A.A.-A.); jlougedo@ucjc.edu (J.H.-L.); 3 HM Hospitals Health Research Institute, 28015 Madrid, Spain

**Keywords:** environmental training, HRC, performance, hypoxic conditions

## Abstract

**Background:** Previous research suggests that resistance training in hypoxia can cause physiological and muscle adaptations. However, this method may not be efficient for individuals who are training to optimize maximal strength and power. **Objective:** This study aimed to investigate the effects of 8 weeks of high-intensity resistance circuit in normobaric hypoxic conditions on maximal and explosive measures of muscle strength in upper and lower limbs. **Methods:** A total of 28 subjects were randomly assigned to either hypoxia (fraction of inspired oxygen [F_I_O_2_] = 15%; HRC_hyp_: n = 15; age: 24.6 ± 6.8 years; height: 177.4 ± 5.9 cm; weight: 74.9 ± 11.5 kg) or normoxia [F_I_O_2_] = 20.9%; HRC_norm_: n = 13; age: 23.2 ± 5.2 years; height: 173.4 ± 6.2 cm; weight: 69.4 ± 7.4 kg) groups. Training sessions consisted of two blocks of three exercises and the training intensity was fixed performed at six repetition maximum. Participants exercised twice weekly for 8 weeks, and upper and lower body power tests were performed before and after the training program. The statistical analysis applied was a two-way analysis of variance with repeated measures and Bonferroni post hoc. **Results:** No significant differences were observed between groups. However, the hypoxia group showed higher intra-group differences in absolute (N) (*F* = 7.97; Δ7.3%; *p* < 0.05; ES = 0.49) and relative (N/Kg) (*F* = 8.34; Δ7.2%; *p* < 0.05; ES = 0.49) maximum push-up force after the training period. **Conclusions:** Hypoxic circuit training may improve a specific upper body performance outcome, but no clear advantage over normoxia was observed.

## 1. Introduction

From a physiological view, reduced inspired oxygen availability lowers arterial O_2_ content and activates oxygen-sensing pathways, primarily via stabilization of hypoxia-inducible factors (HIFs), which regulate transcriptional programs involved in metabolic remodeling and angiogenesis, thereby providing a plausible link to longer-term morphofunctional adaptation [1]. In exercised human skeletal muscle, HIF-1α expression increases after high-intensity or resistance exercise and is accompanied by vascular endothelial growth factor (VEGF) mRNA responses, consistent with the role of transient intramuscular hypoxia in angiogenic signaling [2]. When resistance exercise is performed in normobaric hypoxia, the same external workload can impose a higher internal load (greater physiological strain and metabolic perturbation), which may amplify adaptive signaling and contribute to subsequent functional outcomes [3]. Accordingly, recent interventions and meta-analyses report small, protocol-dependent benefits of normobaric hypoxic resistance training for strength-related outcomes compared with matched normoxia, although findings remain heterogeneous [4,5].

According to resistance exercise, intermittent hypoxic resistance training (IHRT), defined as the combination of resistance exercise with reduced inspired oxygen availability (typically via normobaric hypoxia), has gained substantial attention in both research and applied settings during the last decade [6]. This approach has been proposed to potentiate adaptations to resistance training by modifying the internal physiological milieu during exercise, even when external loads and programming variables are matched. Indeed, several studies have reported greater hypertrophic responses after training under hypoxic conditions compared with equivalent programs performed in normoxia [7,8,9].

In parallel, improvements in maximal strength have also been observed following heavy resistance training in hypoxia, suggesting that hypoxic exposure may enhance performance outcomes beyond purely morphological adaptations in certain contexts [10]. From a mechanistic standpoint, IHRT has been linked to augmented metabolic stress during exercise, which has been repeatedly proposed as a key stimulus contributing to hypertrophy-related signaling and subsequent muscle mass development [11,12]. Beyond morphological adaptations, improvements in maximal strength and explosive performance are largely influenced by neuromuscular factors, including motor unit recruitment and firing behavior, intermuscular coordination, and the ability to rapidly develop force and power [13]. In this context, IHRT may be relevant not only through potential changes in muscle size, but also by altering the fatigue and recovery profile during training, particularly when resistance exercise is performed with short inter-set recoveries and a high density of work [3].

From a mechanistic perspective, exercising under reduced inspired oxygen availability can increase peripheral fatigue and perturb homeostasis for a given external workload, which may necessitate greater recruitment of higher-threshold motor units to maintain force output [14,15]. Additionally, in circuit-based formats, where repeated efforts are performed with limited recovery, reduced oxygen availability may influence the kinetics of recovery between bouts (e.g., phosphagen resynthesis) and thereby place a premium on maintaining force and power under fatigue [16]. These mechanisms provide a plausible link between IHRT and performance outcomes, such as maximal force production and explosive actions, although current evidence remains heterogeneous and any added benefit of hypoxia over matched normoxic training may be outcome- and protocol-specific [3].

Despite this growing body of evidence, most IHRT interventions have been designed with a hypertrophy-oriented focus (i.e., moderate loads, relatively high volumes, and incomplete recoveries) rather than targeting explosive neuromuscular performance [17]. However, resistance training aimed at improving athletic performance frequently incorporates maximal intended velocity and explosive intent, which are key features to elicit task-specific neural adaptations underpinning power production [18]. Consequently, the transferability of the benefits described for hypertrophy-focused IHRT to training modalities emphasizing rapid force production remains uncertain. This is particularly relevant because improvements in explosive performance are often driven by neural and coordinative factors, and the extent to which hypoxia meaningfully interacts with these determinants has not been sufficiently clarified [19].

To date, only a limited number of studies have examined maximal strength- or power-oriented resistance training under hypoxic conditions, either using normobaric hypoxia [20] or moderate terrestrial altitude [9]. For example, Álvarez-Herms et al. [12] reported superior improvements in lower limb anaerobic performance after 4 weeks of training under simulated hypobaric hypoxia compared with normoxia, including enhanced mean countermovement jump height during a 60 s repeated jump test and a reduced fatigue index in the latter phase of the protocol. Likewise, intermittent resistance training at moderate altitude has been shown to affect the force–velocity relationship, isometric strength, and muscle architecture [4]. However, available evidence has predominantly focused on lower limb outcomes, leaving a clear knowledge gap regarding whether similar benefits can be expected for the upper limbs.

Upper limb explosive performance is highly relevant in many sports and occupational tasks. However, arms and legs may not respond identically to hypoxia: compared with the lower limbs, the upper limb musculature generally shows lower O_2_ extraction and different regional perfusion regulation during exercise, which may increase sensitivity to reductions in arterial O_2_ availability [21]. Therefore, under normobaric hypoxia and short recoveries, the upper limbs may experience a relatively greater local deoxygenation/fatigue stimulus for a given external workload, potentially making any additional effect of IHRT more detectable in explosive pushing tasks [22]. Nevertheless, evidence remains limited and limb-specific, so this rationale is presented as a plausible explanation rather than a definitive mechanism.

Thus, the primary aim of this study was to investigate the effects of 8 weeks of high-intensity resistance circuit (HRC) combined with normobaric hypoxia on maximal and explosive measures of muscle strength in upper and lower limbs. In this line, our hypothesis is that, compared to normoxia, a high-intensity strength training circuit in normobaric hypoxia will generate superior improvements in upper limb explosive performance.

## 2. Materials and Methods

### 2.1. Design

To test the effects of 8 weeks of circuit resistance-based training in normoxia and hypoxia on power strength performance, a quasi-experimental single-blinded randomized controlled trial was developed. Participants were allocated in a randomized order, using a specific software (https://www.randomizer.org, (accessed on 1 May 2024)), in two experimental groups: (a) performing 8 weeks of resistance circuit-based training in normobaric hypoxia (HG; FiO_2_ = 15%) and (b) performing the same training in normoxic condition (NG; FiO_2_ = 20.9%). Both groups performed the training sessions in a normobaric chamber (CAT 430, Colorado Altitude training, LOU, CO, USA) placed in the laboratory at an altitude of 475 m above sea level. The system partially reduced oxygen pressure/airflow within the chamber through generators (CAT-12, Colorado Altitude Training, LOU, CO, USA) containing pressurized air serially connected to air filters that allowed oxygen reduction in the airflow input to the chamber. Barometric pressure in the chamber was equivalent to the sea level. The hypoxia group trained with a partial fraction of oxygen (FiO_2_) of 15% (which simulated an altitude of 3400 m, according to the manufacturer’s instructions). However, this altitude was controlled by an electronic device (HANDI+, Maxtec, Salt Lake City, UT, USA).

### 2.2. Participants

Twenty-eight healthy male subjects with at least 4 years of resistance training experience were involved in the experimental process. The subjects were randomly assigned to either the normoxic resistance training group (NG, n = 13) or the hypoxic resistance training group (HG, n = 15). Participants were excluded if they presented any injury or neuromuscular disease. Training and testing protocols were explained, and all participants signed an informed consent to participate in the study. The protocol was reviewed and approved by the Camilo José Cela University bioethics committee (11_24_CrossfitRM; approved on 1 July 2024), following the guidelines of the Declaration of Helsinki, updated at the World Medical Association Assembly in Fortaleza (2013) for research on human subjects. Each participant was assigned a code to maintain their anonymity.

### 2.3. Testing Protocol

**Figure 1:**
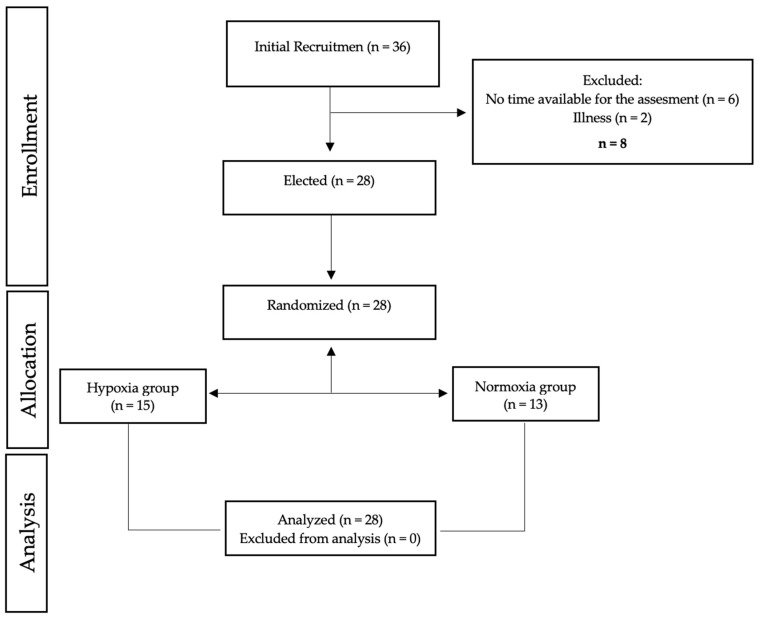
Study design flow-chart.

Three different sessions were used to carry out the testing protocol. On the first day, all subjects visited the laboratory to familiarize themselves with the testing and training protocols the week before starting the training sessions. Pre- and post-tests were carried out 72 h after the last intense workout to allow complete recovery from the training. The testing session included movement jump (CMJ) and push-up test and were carried out 2 h after subjects had a standardized breakfast monitored by a professional nutritionist from the research group. Moreover, all subjects performed the assessments on the same day and at the same time under similar environmental conditions (temperature 20–24 °C, relative humidity 45–55%). After finishing the study, all sessions were repeated in the same order. Figure 1 shows the testing procedures and study design.

### 2.4. Jump Test

CMJ was assessed using a wireless dual force plates system with a sample rate of 1000 Hz (Hawkin Dynamics Inc., Westbrook, ME, USA). All participants were familiarized with this exercise and given the sensitivity of force–time CMJ outcomes to technique, participants were instructed to descend as fast as possible to standardize countermovement timing/stretch-shortening cycle (SSC) strategy use and to keep hands on hips to remove arm swing, which can augment vertical impulse and confound lower limb mechanical outputs. A specific warm-up consisting of 5 min of cycling at 75 w, followed by active stretching and three submaximal jumps, was performed before testing. All participants executed three attempts, and the best result was considered. In relation to rest time between attempts, 2 min were allowed to diminish the effects of fatigue. After this, jump height (cm) and maximal power output (W/Kg) variables were obtained. Through the following equation, h = vi^2^•2g^−1^, jump height (h) was calculated from the take-off vertical velocity (vi). In addition, through the data extracted from the force platform as the product of vertical force by instantaneous vertical velocity of the system’s center of mass, peak power was calculated.

### 2.5. Push-Up Test

Ballistic push-up testing was performed 5 minutes after the CMJ using the same force plates, according to the protocol described by García-Massó et al. [23] (Hawkin Dynamics Inc., Westbrook, ME, USA). Participants were instructed to place their hands in the center of the force plate, shoulder-width apart, and with feet on the floor in a standard push-up position. The participants performed three ballistic push-ups (with only their body weight), separated by 3 min of rest, and the best attempt was analyzed. Previously, 10 push-ups (non-ballistic) and 2 submaximal ballistic push-ups were performed as a task-specific warm-up. An attempt was considered successful when participants achieved a clear flight phase, operationally defined as both hands leaving the force plates, as evidenced by a marked reduction in vertical force below 20 N (or <5% of body weight) for at least 50 ms, followed by a clean bilateral re-contact on the plates. Trials were repeated if a flight phase was not observed, if hand placement changed substantially during the attempt, or if re-contact occurred outside the plates. The ‘best attempt’ was defined as the trial yielding the highest peak vertical force (N), which was the primary kinetic outcome for this test. Relative peak force was calculated by normalizing peak vertical force to body mass (N/kg) and thus was derived from the same selected trial.

### 2.6. Strength Test

6RM loads were determined in the second session, according to Martínez-Guardado et al. [2], to evaluate muscular strength, but also because the training protocol would be performed with such loads. The order of the exercises was the same as that used in the training protocol: bench press, leg extension, front lat pulldown, deadlift, elbow flexion (preacher curl), and ankle extension. To calculate the 6RM loads, subjects performed 3 sets of each exercise using the following sequence: 10 repetitions at 50% of the perceived 6RM, 1 min of rest, 8 repetitions at 75% of estimated 6RM, 2 min of rest, and 1 set of the exercises to volitional fatigue at 100% of estimated 6RM. If a subject performed +1 repetition, the training load was adjusted by approximately +2.5, and if a subject completed +2 repetitions, the training load was adjusted by 65% [24]. The subjects were allowed to do 5 attempts at maximum with 5 min of rest between each attempt. The rest period between exercise was 5 min. Before testing, a warm-up consisting of 5 min of cycling at 75 w, followed by active stretching and 10 repetitions at 50% of the perceived 1RM for each exercise, was performed.

### 2.7. Training Program Procedure

**Figure 2:**
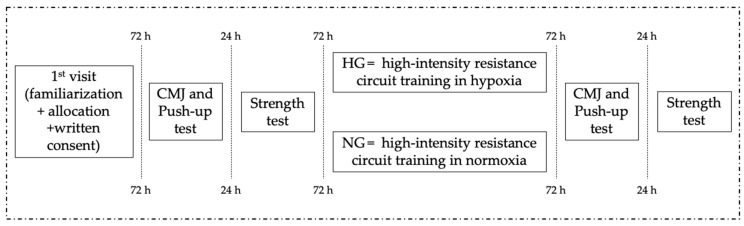
Research design.

Participants performed a high-intensity resistance circuit (HRC) training program [7], which consisted of two short circuits (blocks) with 35 s of rest between exercises (that allowed enough time to move safely from one exercise to the next), a 3 min rest between each set of three exercises within a block, and a 5 min rest between blocks. Block 1 consisted of bench press, leg extension, and front pulldown exercises. Block 2 involved deadlift, elbow flexion (preacher curl), and ankle extension (calf raise). Participants performed 3 sets of each exercise of the block in the same order that has previously been described. Block 1 always preceeded Block 2 in each HRC training session. Two training sessions per week were performed by each participant. There were more than 48 h of rest between each training session (Figure 2).

A general warm-up in normoxia prior to the workout session was performed, which included 5 min of submaximal running at 8.5 km/h, followed by 5 min of active stretching of all major muscle groups. Also, a specific warm-up, consisting of 3 sets of the exercises of the first block, was completed using the following sequence: 10 repetitions at 50% of 6RM for each exercise, 1 min rest, 8 repetitions at 75% of 6RM, 2 min rest, and repetitions to failure with the 6RM load. This specific warm-up was used to ensure that participants lifted loads that allowed for only six repetitions (∼85–90% of 1RM). If necessary, the training load was adjusted by ±2.5% if a subject performed ±1 repetition and by ±5% if a subject completed ±2 repetitions. The specific warm-up and the training session were performed according to the group assignment, either in hypoxia or in normoxia. All training sessions and rest periods were strictly controlled by an experienced lifter. Table 1 shows the training program performed during the 8 weeks of the study.

### 2.8. Statistical Analysis

The statistical analysis was carried out with SPSS for Windows statistical package (version 24.0; SPSS, Inc., Chicago, IL, USA). The Shapiro–Wilk test was applied in order to verify a normal distribution of data, and Levene’s test was used to assess the homoscedasticity of variance. After this, a two-way analysis of variance with repeated measures and Bonferroni post hoc test was used to investigate the main effects and the interaction between group factor (hypoxia vs. normoxia) and time factor (pre-training vs. post-training). The effect size (ES) of the intervention was calculated using Cohen’s guidelines and were classified as small (>0.2), moderate (>0.6), large (>1.2), and very large (>2.0) [25]. For all procedures, a level of *p* ≤ 0.05 was selected to indicate statistical significance. The statistical power was calculated at posteriori by effect size. The sample size (n = 28) was large enough to obtain an effect size value between 1.06 and 1.36, considering a sensitivity to detect real effects of change between 80 and 95%.

## 3. Results

Table 2 shows the sociodemographic, anthropometric, and body composition information of the participants.

**Figure 3:**
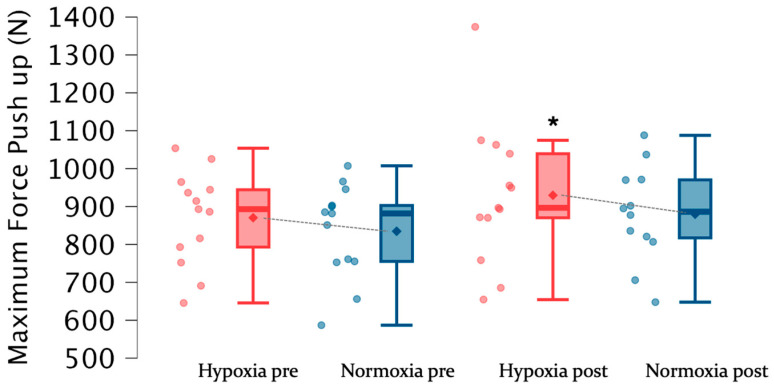
Values of maximum force (N) per subject and group pre- and post-training program. * = refers to intra-group differences; • = individual response in the variable for the hypoxia group; • = individual response in the variable for the normoxia group; ♦ = refers to the mean value for the variable for the hypoxia group; ♦ = refers to the mean value for the variable for the normoxia group.

**Figure 4:**
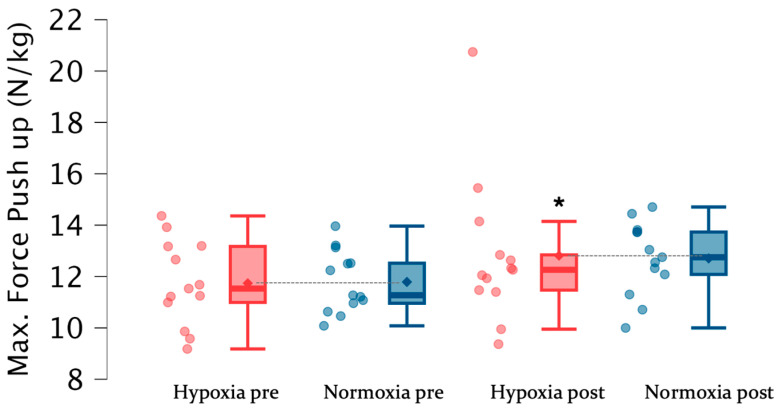
Values of maximum relative force (N/Kg) per subject and group pre- and post-training program. * = refers to intra-group differences; • = individual response in the variable for the hypoxia group; • = individual response in the variable for the normoxia group; ♦ = refers to the mean value for the variable for the hypoxia group; ♦ = refers to the mean value for the variable for the normoxia group.

Table 3 shows the results of push-up and jump test before and at the end of the training program in both groups. There were no significant intra-group differences between pre- and post-training CMJ values in both groups. Also, no significant differences were observed between groups before and after the training program. However, after training, higher absolute (N) (*F* = 7.97; Δ7.3%; *p* < 0.05; ES = 0.49) and relative (N/Kg) (*F* = 8.34; Δ7.2%; *p* < 0.05; ES = 0.49) maximum (Figure 3) and relative (Figure 4) push-up force was observed in HG compared to pre-training values.

## 4. Discussion

To our knowledge, this is one of the first studies examining the effects of 8 weeks of high-intensity resistance circuit training performed under normobaric hypoxia (FiO_2_ ≈ 15%) versus normoxia on both maximal and explosive strength outcomes in the upper and lower body. The main finding was that the training program was associated with small improvements in selected upper body strength outcomes, while lower-body explosive performance (CMJ height/power) did not clearly change. Importantly, the normoxia group showed comparable trends, and we did not observe significant between-group differences, suggesting that any additional benefit of hypoxia, under the present protocol, is likely modest and outcome-specific. Collectively, these results are consistent with the broader literature, indicating that resistance training performed in hypoxia may improve certain strength-related outcomes, although effects are heterogeneous and appear highly dependent on methodological variables (e.g., exercise selection, intensity, rest intervals, set configuration, total volume, and hypoxic dose) [26].

Regarding upper body performance, the hypoxia group showed a significant increase in peak and relative force during the ballistic push-up; however, the normoxia group exhibited a similar magnitude change that approached significance. Therefore, this finding should be interpreted primarily as a training-related adaptation rather than evidence of a clear hypoxia-specific advantage, especially given the absence of significant between-group differences. Importantly, generalizability is limited because the bench press was the only exercise in the program that substantially targeted horizontal pushing musculature. As the ballistic push-up is a reliable measure of upper body strength/power and is closely related to bench press performance, improvements may partly reflect task-specific transfer and neuromuscular learning that would be expected in both conditions [27,28]. Mechanistically, normobaric hypoxia can increase acute internal load and physiological strain for a given external workload [3], which could theoretically influence neural drive and motor unit behavior during repeated high-intensity efforts. Nevertheless, comparable high-resistance circuit interventions have reported largely similar neuromuscular adaptations in hypoxia and normoxia, including H-reflex-related indices, and meta-analytic evidence suggests that any additional strength benefit of hypoxic resistance training over matched normoxic training is generally small and protocol-dependent [29,30].

Regarding lower-body outcomes, CMJ height and power did not show clear improvements following hypoxic traning, and no significant between-group differences were observed. This is broadly consistent with the mixed evidence on IHRT for jump-related outcomes, where changes in CMJ performance are often small or unclear when training loads and programs are matched between hypoxia and normoxia [17,31]. Notably, Inness et al. [10] reported a small increase in CMJ peak power after heavy IHRT, yet the between-group difference remained unclear, reinforcing the interpretation that hypoxia does not reliably add benefit for explosive lower-body outcomes.

Contrary to upper body, a major design-related limitation that likely explains our null CMJ findings is the lack of specific lower body explosive training content. Given the well-established principle of training specificity, CMJ improvements typically require a sufficiently specific stimulus emphasizing high movement velocity and/or plyometric–SSC demands. In this line, previous research has shown meaningful CMJ adaptations within ~6–8 weeks when training includes plyometric/complex or power-oriented elements [32,33]. Therefore, the absence of CMJ changes in the present study is more plausibly attributed to limited transfer from a circuit format not designed to target explosive lower limb mechanics rather than to insufficient intervention duration per se. Finally, our findings align with current evidence indicating that outcomes of hypoxic resistance training are highly sensitive to programming and hypoxic-dose, which may determine whether adaptations are more ‘strength-like’ versus ‘power-like’ [5,34].

In practical terms, high-intensity resistance circuit training performed under moderate normobaric hypoxia (FiO_2_ ≈ 15%) appears feasible and does not seem to impair neuromuscular adaptations compared with the same program in normoxia. However, the circuit was not designed as a power-focused intervention, particularly for the lower body, and did not include explosive/ballistic lower limb exercises; thus, this mismatch between the training stimulus and the selected power outcomes is a major limitation and likely explains the null lower body findings. Therefore, if improving power is the primary goal, power-specific exercises and loading strategies should be prioritized, with hypoxia considered only as a potential adjunct.

## 5. Conclusions

Overall, 8 weeks of high-intensity resistance circuit training performed under normobaric hypoxia was associated with a small improvement in one upper body strength outcome, while other outcomes showed trivial-to-small changes. Importantly, the normoxia group demonstrated similar trends and no significant between-group differences were observed; therefore, the added value of hypoxic exposure over matched normoxic training remains unclear in the present study. Lower-body explosive performance did not clearly change, which is likely related to the lack of lower limb power-specific training content, highlighting the principle of training specificity. Future studies using larger samples and power-oriented lower-body exercises are needed to determine whether hypoxia can provide incremental benefits for strength and power adaptations.

## Figures and Tables

**Table 1:** Training program performed during the study.

Week	1	2	3	4	5	6	7	8
Set	2	3	3	2	3	3	4	2
Repetitions	6	6	6	6	6	6	6	6
% 6RM	90	95	100	100	100	100	100	100

RM: Repetition maximum.

**Table 2:** Participants’ characteristics.

Parameters	HG (Mean ± SD)	NG (Mean ± SD)
Age (years)	24.61 ± 6.79	23.24 ± 5.16
Height (m)	177.42 ± 5.91	173.44 ± 6.21
Weight (kg)	74.86 ± 11.45	69.44 ± 7.35
Body Mass Index	25.90 ± 1.30	23.20 ± 2.49
Lean Body Mass (kg)	60.48 ± 7.87	56.40 ± 5.04
Fat Mass (%)	14.47 ± 5.51	16.28 ± 5.54

**Table 3:** CMJ and push-up values for hypoxia and normoxia groups (mean ± SD).

		Pre-Training		Post-Training					95% CI for Difference			Group Effect	Time Effect	Group × Time
		Mean	SD	Mean	SD	ES	*p *	η^2^_p_	Mean Difference	Lower Bound	Upper Bound			
Height Jump (cm)	HG	29.6	5.5	29.7	4.7	0.02	0.901	0.024	0.1	−1.5	1.6	0.447	0.801	0.939
	NG	31.2	6.3	31.3	5.8	0.03	0.816		0.1	−1.7	1.4			
Maximum Power CMJ (w)	HG	3547.5	774.9	3497.5	691.8	0.06	0.517	0.002	−50.0	−206.8	106.8	0.847	0.824	0.487
	NG	3461.7	506.2	3487.6	540.3	0.05	0.737		25.8	−131.0	182.6			
Maximum Force Push-Up (N)	HG	871.0	124.0	934.7	209.6	0.49	0.033 *	0.023	63.7	5.5	121.9	0.457	0.009	0.713
	NG	835.3	124.1	884.2	142.5	0.37	0.096		48.8	−9.3	107.0			
Maximum Force Push-Up (N/kg)	HG	11.7	1.7	12.6	2.9	0.49	0.034 *	0.003	0.9	0.1	1.6	0.968	0.008	0.772
	NG	11.8	1.2	12.5	1.4	0.53	0.079		0.7	−0.1	1.5			

HG: Hypoxia group; NG: normoxia group; * = refers to intra-group differences; ES: Cohen’s effect size; η^2^_p_: between-group effect sizes.

## Data Availability

The data that support the findings of this study are available on request from the corresponding author. The data are not publicly available due to privacy or ethical restrictions.

## References

[1] Bakleh M.Z., Al Haj Zen A. (2025). The Distinct Role of HIF-1α and HIF-2α in Hypoxia and Angiogenesis. Cells.

[2] Aragón-Vela J., Casuso R.A. (2025). Effect of Hypoxia-Inducible Factor 1 on Vascular Endothelial Growth Factor Expression in Exercised Human Skeletal Muscle: A Systematic Review and Meta-Analysis. Am. J. Physiol.-Cell Physiol..

[3] Allsopp G.L., Britto F.A., Wright C.R., Deldicque L. (2024). The Effects of Normobaric Hypoxia on the Acute Physiological Responses to Resistance Training: A Narrative Review. J. Strength Cond. Res..

[4] Benavente C., Padial P., Scott B.R., Almeida F., Olcina G., Pérez-Regalado S., Feriche B. (2024). Strength and Muscle Mass Development after a Resistance-Training Period at Terrestrial and Normobaric Intermittent Hypoxia. Pflug. Arch.—Eur. J. Physiol..

[5] Wang H., Tuerhongjiang M., Zeng Z., Wang Y., Liu J., Liu R. (2025). The Effects of Normobaric Hypoxic Resistance Training on Muscle Strength in Healthy Adults. Eur. J. Appl. Physiol..

[6] Scott B.R., Slattery K.M., Sculley D.V., Dascombe B.J. (2014). Hypoxia and Resistance Exercise: A Comparison of Localized and Systemic Methods. Sports Med..

[7] Martínez-Guardado I., Ramos-Campo D.J., Olcina G.J., Rubio-Arias J.A., Chung L.H., Marín-Cascales E., Alcaraz P.E., Timón R. (2019). Effects of High-Intensity Resistance Circuit-Based Training in Hypoxia on Body Composition and Strength Performance. Eur. J. Sport Sci..

[8] Martínez-Guardado I., Sánchez-Ureña B., Camacho-Cardenosa A., Camacho-Cardenosa M., Olcina G., Timón R. (2020). Effects of Strength Training under Hypoxic Conditions on Muscle Performance, Body Composition and Haematological Variables. Biol. Sport.

[9] Morales-Artacho A.J., Padial P., García-Ramos A., Pérez-Castilla A., Argüelles-Cienfuegos J., De la Fuente B., Feriche B. (2018). Intermittent Resistance Training at Moderate Altitude: Effects on the Force-Velocity Relationship, Isometric Strength and Muscle Architecture. Front. Physiol..

[10] Inness M.W., Billaut F., Walker E.J., Petersen A.C., Alice J., Aughey R.J. (2016). Heavy Resistance Training in Hypoxia Enhances 1RM Squat Performance. Front. Physiol..

[11] Scott B.R., Slattery K.M., Dascombe B.J. (2015). Intermittent Hypoxic Resistance Training: Is Metabolic Stress the Key Moderator?. Med. Hypotheses.

[12] Chycki J., Czuba M., Gołaś A., Zając A., Fidos-Czuba O., Młynarz A., Smółka W. (2016). Neuroendocrine Responses and Body Composition Changes Following Resistance Training under Normobaric Hypoxia. J. Hum. Kinet..

[13] Rong W., Geok S.K., Samsudin S., Zhao Y., Ma H., Zhang X. (2025). Effects of Strength Training on Neuromuscular Adaptations in the Development of Maximal Strength: A Systematic Review and Meta-Analysis. Sci. Rep..

[14] Simpson C.W.C., Walter J., Gieseg S.P., Lackner S., Holasek S., Hamlin M.J. (2025). Central and Peripheral Nervous System Activity and Muscle Oxygenation in Athletes during Repeated-Sprint Exercise in Normoxia and Normobaric Hypoxia. J. Sports Sci..

[15] Bondi D., Valli G., Santangelo C., Annarumma S., Pietrangelo T., Fulle S., Verratti V. (2025). Non-Invasive Motor Unit Analysis Reveals Specific Responses during Maximal Muscle Contraction under Normobaric Hypoxia. Pflug. Arch.—Eur. J. Physiol..

[16] Maliszewski K., Feldmann A., McCully K.K., Julian R. (2024). A Systematic Review of the Relationship between Muscle Oxygen Dynamics and Energy Rich Phosphates. Can NIRS Help?. BMC Sports Sci. Med. Rehabil..

[17] Feriche B., García-Ramos A., Morales-Artacho A.J., Padial P. (2017). Resistance Training Using Different Hypoxic Training Strategies: A Basis for Hypertrophy and Muscle Power Development. Sports Med.—Open.

[18] Buckthorpe M., Erskine R.M., Fletcher G., Folland J.P. (2015). Task-Specific Neural Adaptations to Isoinertial Resistance Training. Scand. J. Med. Sci. Sports.

[19] Cormie P., McGuigan M.R., Newton R.U. (2011). Developing Maximal Neuromuscular Power: Part 1—Biological Basis of Maximal Power Production. Sports Med..

[20] Álvarez-Herms J., Julià-Sánchez S., Corbi F., Pagès T., Viscor G. (2014). Anaerobic Performance after Endurance Strength Training in Hypobaric Environment. Sci. Sports.

[21] Calbet J.A.L., Holmberg H.-C., Rosdahl H., van Hall G., Jensen-Urstad M., Saltin B. (2005). Why Do Arms Extract Less Oxygen than Legs during Exercise?. Am. J. Physiol. Regul. Integr. Comp. Physiol..

[22] Willis S.J., Millet G.P., Borrani F. (2020). Insights for Blood Flow Restriction and Hypoxia in Leg versus Arm Submaximal Exercise. Int. J. Sports Physiol. Perform..

[23] García-Massó X., Colado J.C., González L.M., Salvá P., Alves J., Tella V., Triplett N.T. (2011). Myoelectric Activation and Kinetics of Different Plyometric Push-up Exercises. J. Strength Cond. Res..

[24] Blazevich A.J., Gill N.D., Zhou S. (2006). Intra- and Intermuscular Variation in Human Quadriceps Femoris Architecture Assessed in Vivo. J. Anat..

[25] Hopkins W.G., Marshall S.W., Batterham A.M., Hanin J. (2009). Progressive Statistics for Studies in Sports Medicine and Exercise Science. Med. Sci. Sports Exerc..

[26] Ramos-Campo D.J., Scott B.R., Alcaraz P.E., Rubio-Arias J.A. (2018). The Efficacy of Resistance Training in Hypoxia to Enhance Strength and Muscle Growth: A Systematic Review and Meta-Analysis. Eur. J. Sport Sci..

[27] Wang R., Hoffman J.R., Sadres E., Bartolomei S., Muddle T.W., Fukuda D.H., Stout J.R. (2017). Evaluating Upper-Body Strength and Power from a Single Test: The Ballistic Push-Up. J. Strength Cond. Res..

[28] Van Den Tillaar R., Ball N. (2020). Push-Ups Are Able to Predict the Bench Press 1-RM and Constitute an Alternative for Measuring Maximum Upper Body Strength Based on Load-Velocity Relationships. J. Hum. Kinet..

[29] Ramos-Campo D.J., Martínez-Guardado I., Rubio-Arias J.A., Freitas T.T., Othalawa S., Andreu L., Timón R., Alcaraz P.E. (2021). Muscle Architecture and Neuromuscular Changes after High-Resistance Circuit Training in Hypoxia. J. Strength Cond. Res..

[30] Benavente C., Schoenfeld B.J., Padial P., Feriche B. (2023). Efficacy of Resistance Training in Hypoxia on Muscle Hypertrophy and Strength Development: A Systematic Review with Meta-Analysis. Sci. Rep..

[31] Törpel A., Peter B., Schega L. (2020). Effect of Resistance Training under Normobaric Hypoxia on Physical Performance, Hematological Parameters, and Body Composition in Young and Old People. Front. Physiol..

[32] Ma S., Xu Y., Xu S. (2025). Effects of Physical Training Programs on Healthy Athletes’ Vertical Jump Height: A Systematic Review with Meta-Analysis. J. Sports Sci. Med..

[33] Sun R., Qu H., Zhang Y., Wang H. (2025). The Effect of Complex Training on Strength, Counter Movement Jump and Change of Direction Skills in Female Junior Table Tennis Players. Sci. Rep..

[34] Benavente C., Feriche B. (2025). The Influence of Specific Resistance Training Methodological Prescription Variables on Strength Development under Hypoxic Conditions: A Systematic Review and Meta-Analysis. J. Sports Sci..

